# A training ground Lay out improves rehabilitation after trauma surgery: A Fast Track Policy: F Rubba

**DOI:** 10.1093/eurpub/ckac131.011

**Published:** 2022-10-25

**Authors:** G Balato, R Palladino, E Montella, L Diana, A Coviello, E Festa, A Iervolino, F Rubba, M Mariconda, M Triassi

**Affiliations:** 1 Othopedics Surgery Units, AOU Federico II, Naples, Italy; 2 Public Health Department, AOU Federico II, Naples, Italy; 3 University Hospital, AOU Federico II, Naples, Italy

## Abstract

Fracture patients are frail and have high mortality. We investigated whether introducing a fast-track strategy during post-surgery care and including early rehabilitation protocols may shorten the length of hospital stay (LOS) while improving the overall clinical effectiveness. A training ground was built inside the inpatient area dedicated to trauma settings. Usual postoperative care consists of immobilization during the first day, but patients may start rehabilitation earlier, 24 hours after the surgical procedure, with a fast-track strategy. In general, gait speed, step length, and self-assessment in terms of mobility improve significantly in the first six postoperative weeks in fracture patients. As delayed postoperative mobility during hospitalization was observed, the established training ground may help with this concern. The expert physiotherapist may contribute to ameliorating the indicators showing great potential in postoperative rehabilitation regardless of fracture pattern. The primary outcome was postoperative physical functioning. Secondary outcomes included the patient’s assessment of therapeutic effect (overall improvement), perceived pain intensity, health services utilization, treatment side effects, and adverse events. Data were analyzed by univariate analysis and binary logistic regression showing a reduction of LOS of almost three days. Further, the optimized hip fracture program reduced the rate of in-hospital postoperative complications and mortality. Adding to the schedule, some PROMPTS (Patient-reported outcome measures) could further integrate the patient empowerment perspective into the quality set of values. For this reason, ‘fast track’ may define a crucial policy able to guarantee rapid rehabilitation, becoming a key factor to achieving a good clinical effect. Fast-track rehabilitation facilitates a shortened hospital stay and cost-saving and can be used to optimize the patient’s condition before admission to a rehabilitation facility

**Key messages:**

• Early rehabilitation protocols may shorten the length of hospital stay (LOS) while improving the overall clinical effectiveness.

• Human factors and patients empowerments may help.

